# Programmed cell death in type II neuroblast lineages is required for central complex development in the *Drosophila *brain

**DOI:** 10.1186/1749-8104-7-3

**Published:** 2012-01-18

**Authors:** Yanrui Jiang, Heinrich Reichert

**Affiliations:** 1 Biozentrum, University of Basel, CH-4056 Basel, Switzerland

## Abstract

**Background:**

The number of neurons generated by neural stem cells is dependent upon the regulation of cell proliferation and by programmed cell death. Recently, novel neural stem cells that amplify neural proliferation through intermediate neural progenitors, called type II neuroblasts, have been discovered, which are active during brain development in *Drosophila*. We investigated programmed cell death in the dorsomedial (DM) amplifying type II lineages that contribute neurons to the development of the central complex in *Drosophila*, using clonal mosaic analysis with a repressible cell marker (MARCM) and lineage-tracing techniques.

**Results:**

A significant number of the adult-specific neurons generated in these DM lineages were eliminated by programmed cell death. Programmed cell death occurred during both larval and pupal stages. During larval development, approximately one-quarter of the neuronal (but not glial) cells in the lineages were eliminated by apoptosis before the formation of synaptic connectivity during pupal stages. Lineage-tracing experiments documented the extensive contribution of intermediate neural progenitor-containing DM lineages to all of the major modular substructures of the adult central complex. Moreover, blockage of apoptotic cell death specifically in these lineages led to prominent innervation defects of DM-derived neural progeny in the major neuropile substructures of the adult central complex.

**Conclusions:**

Our findings indicate that significant neural overproliferation occurs normally in type II DM lineage development, and that elimination of excess neurons in these lineages through programmed cell death is required for the formation of correct neuropile innervation in the developing central complex. Thus, amplification of neuronal proliferation through intermediate progenitors and reduction of neuronal number through programmed cell death operate in concert in type II neural stem-cell lineages during brain development.

## Background

The *Drosophila *central brain is a highly complex neural structure comprising several tens of thousands of neural cells that are organized into the intricate synaptic circuitry of the neuropile. The neurons of the brain are generated during development by a remarkably small set of approximately 100 bilaterally symmetrical pairs of neural stem-cell-like primary progenitors referred to as 'neuroblasts' [1,2]. These neuroblasts undergo two phases of neurogenesis; the first takes place during embryogenesis, and the second occurs during the post-embryonic larval phase [3,4]. During embryonic neurogenesis, the brain neuroblasts produce the primary neurons of the larval brain. After a quiescent phase, most of the same neuroblasts restart their proliferative activity, and produce the adult-specific secondary neurons of the adult brain during larval development. These secondary neurons, which represent approximately 95% of the neurons present in the adult brain, form synaptic interconnections during subsequent pupal development.

Recent work has shown that the neuroblasts of the brain can be divided into two classes: type I and type II. Most of the neuroblasts in the fly brain are type I neuroblasts, which generate their neural progeny via a non-self-renewing intermediate progenitor called a ganglion mother cell (GMC), which divides only once to give rise to two post-mitotic cells, either neurons or glial cells [5-8]. By contrast, eight identified type II brain neuroblast pairs generate their progeny through self-renewing intermediate neural progenitors (INPs), which have features of transit amplifying cells. Because an INP undergoes several rounds of proliferative cell divisions, each of which results in self-renewal of the INP and in the generation of a GMC that produces two neural progeny, a marked amplification of proliferation occurs in type II lineages [9-11]. Thus, whereas most type I neuroblasts generate neural lineages consisting of approximately 100 to 150 adult-specific neurons, type II neuroblasts typically give rise to adult-specific neural lineages that are 3 to 5 times larger (range 370 to 580 [9]).

Recent studies have indicated that the amplifying type II neuroblast lineages primarily contribute neural cells to a complex unpaired neuropile center in the adult brain, called the central complex [12-14]. The central complex is located in the midline of the protocerebrum, and comprises several thousand neurons, corresponding to approximately 50 cell types that project into several major modular compartments, including the ellipsoid body (EB), fan-shaped body (FB), noduli (NO), and protocerebral bridge (PB), as well as associated accessory regions. A comprehensive neuroblast lineage-based analysis indicates that most of the adult-specific neurons in the central complex derive from 10 identified neuroblast lineages [15]. Prominent among these are the type II neuroblast lineages, notably the six type II lineages located at the posterior dorsomedial edge of the brain hemispheres termed DM1 to DM6 [9,12]. For example, four of these DM lineages (DM1 to DM4) generate the central complex small-field neurons that link the major neuropile modules of the central complex [12,15]. Thus, a relatively small number of amplifying type II neuroblasts generates a remarkably large number of adult-specific central complex neurons during a relatively short period of post-embryonic development.

Although the amplification of neural stem-cell proliferation through INPs can generate large numbers of progeny in a short time, it is sensitive to dysregulation, which can result in overproliferation and tumorigenesis. Thus, tight control of proliferation in both the neuroblast and the INP is essential to prevent this type of dysregulation [16]. In addition to this restriction of the developmental potential of progenitors, programmed cell death also plays an important role in regulating both the number of proliferating progenitors and the number of their post-mitotic neural progeny. Prominent programmed cell death of type I neuroblasts and neurons has been documented in the brain and ventral nerve cord during embryonic and post-embryonic neurogenesis [17-23]. Moreover, lineage-specific programmed cell death has recently been reported in post-embryonic development of the Engrailed-expressing neuronal lineages in the brain, and in identified neuroblast lineages in which cell death is limited in specific hemilineages [24-26]. However, nothing is currently known about a potential role for programmed cell death in the amplifying type II neuroblast lineages. Given the large number of neurons produced in these lineages and their susceptibility to overproliferation, programmed cell death could play an important regulatory role in their development.

In this study, we used clonal mosaic analysis with a repressible cell marker (MARCM) [27] and lineage-tracing techniques to investigate programmed cell death in the amplifying DM lineages that contribute neurons to central complex development. We found that programmed cell death occurred in the amplifying DM lineages during both larval and pupal stages. During larval development, approximately one-quarter of the neuronal (but not glial) cells in the lineages were eliminated by apoptosis before the formation of synaptic connectivity during pupal stages. Lineage-tracing experiments document the extensive contribution of INP-containing DM lineages to all of the major modular neuropile substructures of the adult central complex. Moreover, blockage of apoptotic cell death specifically in these lineages led to prominent innervation defects of DM-derived neural progeny in all the major substructures of the adult central complex neuropile. Our findings indicate that significant neural overproliferation normally occurs in DM lineage development, and that elimination of excess neurons in these lineages through programmed cell death is required for the formation of correct neuropile innervation in the developing central complex.

## Results

### Programmed cell death occurs in DM lineages during post-embryonic development

Six of the eight type II neuroblasts present in each half of the central brain are located at the posterior dorsomedial edge of the hemisphere. These six neuroblasts and their respective lineages have been referred to as DM1 to DM6 by Bello *et al*. [9], and they tentatively correspond to the DPMm1 (DM1), DPMpm1 (DM2), DPMpm2 (DM3), CM4 (DM4), CM3 (DM5), and CM1 (DM6) lineages neuroanatomically defined by Pereanu *et al*. [15]. A large number of neurons in these DM lineages makes major contributions to the central complex [12,13,15]. Because they are relatively easy to identify, most investigations of type II neuroblast lineages have focused on DM1 to DM6.

To determine if some of the neurons generated in these DM1 to DM6 lineages might be eliminated by lineage-specific programmed cell death, we combined a lineage-tracing method with targeted apoptosis block. For DM-specific lineage tracing, we used the *erm-Gal4^R09D11 ^*driver together with *UAS-flp actin>CD2>Gal4*, resulting in FLP-out clones that permanently expressed GAL4 in all of the neural progeny of all DM lineages [13]. In the larval central brain, the *erm-Gal4^R09D11 ^*driver alone specifically targets all mature INPs and their young progeny; however, it does not target all of these neural progeny in pupal stages or in the adult, thus making this type of lineage tracing necessary [13,28-30]. A DM-specific apoptosis block was attained by combination of this *erm-Gal4^R09D11^*-based lineage-tracing method with *UAS-p35*; the latter expresses the baculovirus anti-apoptotic protein P35 [31]. DM-specific labeling was attained by *UAS-mCD8::GFP*, and general synaptic neuropile labeling by the widely used nc82 monoclonal antibody [32-34].

**Figure 1:**
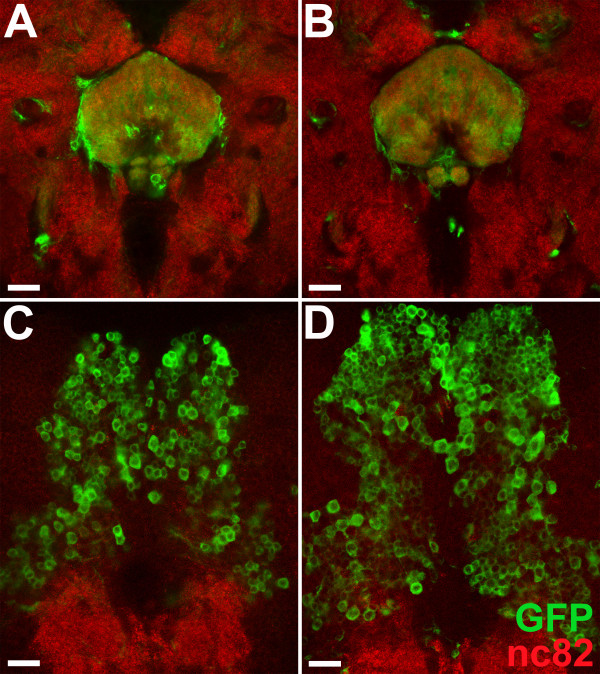
**An increased number of neural cell somata is present in apoptosis-blocked dorsomedial (DM) lineages**. **(A, B) **Neuropile labeled by nc82 and DM lineage-derived cells labeled with green fluorescent protein (GFP) in (A) wild-type flies and (B) DM lineage-specific apoptosis-blocked flies. Single confocal sections at the level of the fan-shaped body of the central complex are shown. **(C, D) **Neuropile labeled with nc82 and DM lineage-derived cell bodies labeled with GFP in (C) wild-type and (D) DM lineage-specific apoptosis-blocked flies. Single confocal sections at the level of the posterior dorsal midline are shown. Note the marked increase in the number of labeled DM lineage-derived cell somata in the apoptosis-blocked flies compared with wild-type. (A) and (C) are from the same preparation, as are (B) and (D), and both preparations are from pupae 48 hours after puparium formation. Scale bars = 20 μm. Genotypes: **(A, C) ***UAS-flp; erm-Gal4^R09D11 ^act>CD2>Gal4 UAS-mCD8::GFP ***(B, D) ***UAS-flp; UAS-p35; erm-Gal4^R09D11 ^act>CD2>Gal4 UAS-mCD8::GFP*.

Analysis of the central brain neuropile in wild-type versus DM-specific apoptosis-blocked flies did not reveal gross neuroanatomical changes at the level of the principal central complex compartments (but see further detailed analysis below). Thus, major substructures of the central complex such as the FB and NO were present and comparable in both cases. Moreover, in both cases DM lineage-derived cells contributed to the central complex neuropile. This is apparent in single confocal sections taken at the level of the FB and NO (Figure 1A, B). However, a marked increase in the number of labeled DM lineage-derived cell somata was seen in the apoptosis-blocked animals compared with the wild-type animals (Figure 1C, D). This increase was especially apparent in single confocal sections taken at the level of the posterior dorsal midline of the brain (Figure 1C, D). These results suggest that a significant amount of programmed cell death occurs in the adult-specific neurons of DM lineages during post-embryonic brain development.

**Figure 2:**
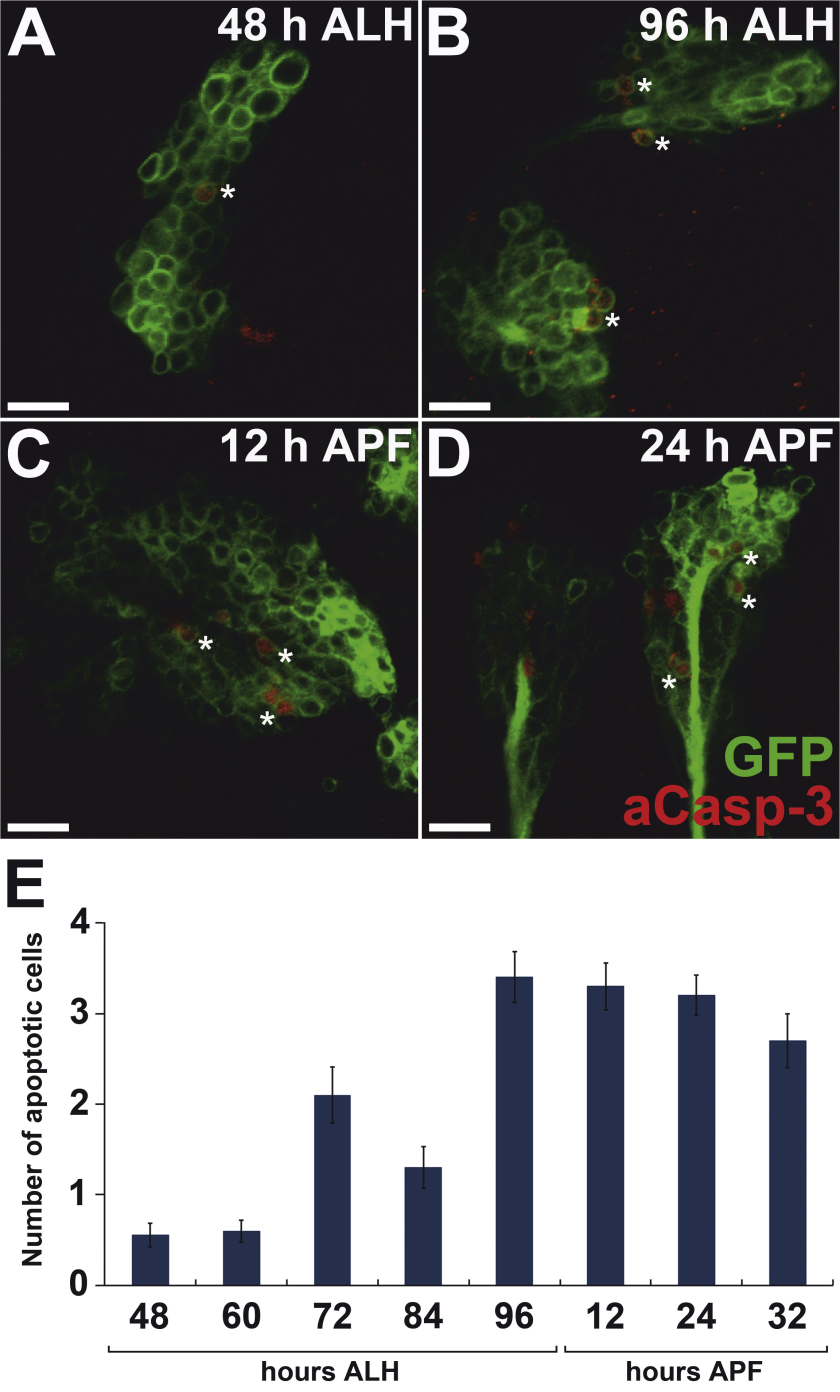
**Programmed cell death in dorsomedial (DM) lineages occurs during both larval and pupal development**. **(A-D) **DM lineage cells expressing activated Caspase-3 (aCasp-3) at different times during post-embryonic development. Single confocal sections show activated Caspase-3 (red) and DM lineage cells labeled with mCD8::GFP (green) at (A) 48 and (B) 96 hours after larval hatching (ALH), and (C) 12 and (D) 24 hours after puparium formation (APF). Asterisks indicate cells undergoing apoptosis. **(E) **Average number of observed apoptotic cells per DM lineage at different stages during larval and pupal development (error bars represent the standard error). For each time point, more than 36 DM lineages were examined. Scale bars = 10 μm. Genotype: (A-D) *UAS-mCD8::GFP; erm-Gal4^R09D11^*.

To confirm the existence of programmed cell death in the six DM lineages, we used *erm-Gal4^R09D11^*-based labeling of the DM lineages together with immunostaining with an antibody that recognizes activated Caspase-3, and labels cells acutely undergoing apoptosis [35]. These experiments showed that cells expressing activated Caspase-3 are found in the wild-type DM lineages during both larval and pupal development (Figure 2A-D, asterisks). For quantification, we determined the total number of cells in DM lineages expressing activated Caspase-3 at 48, 60, 72, 84, and 96 hours after larval hatching (ALH) and at 12, 24, and 32 hours after puparium formation (APF). These experiments identified a level of apoptotic activity of less than one immunoreactive cell per lineage at 48 and 60 hours ALH, which increased to a level of approximately three immunoreactive cells per lineage at 96 hours ALH, and remained at this level throughout the first third of pupal development (Figure 2E) (note that these numbers reflect the cells that were actively undergoing the rapid process of apoptosis at the time of observation, thus at any given instant during the late larval and early pupal development, an average of three cells were undergoing apoptosis in each DM lineage). Furthermore, programmed cell death occurred in all six DM lineages, as activated Caspase-3 was seen in cells of all DM lineages at any given time point. Previous studies have shown that most of the cells in the DM lineages are generated between 72 and 96 hours ALH, and that axonal sprouting, terminal arborization, and formation of synaptic contacts occurs during early pupal development [9,12,15]. This suggests that neural cells in the DM lineages are eliminated by programmed cell death both before and during the formation of synaptic connections in the central brain neuropile.

### Identification and quantification of apoptotic cells in DM lineages during larval development

**Figure 3:**
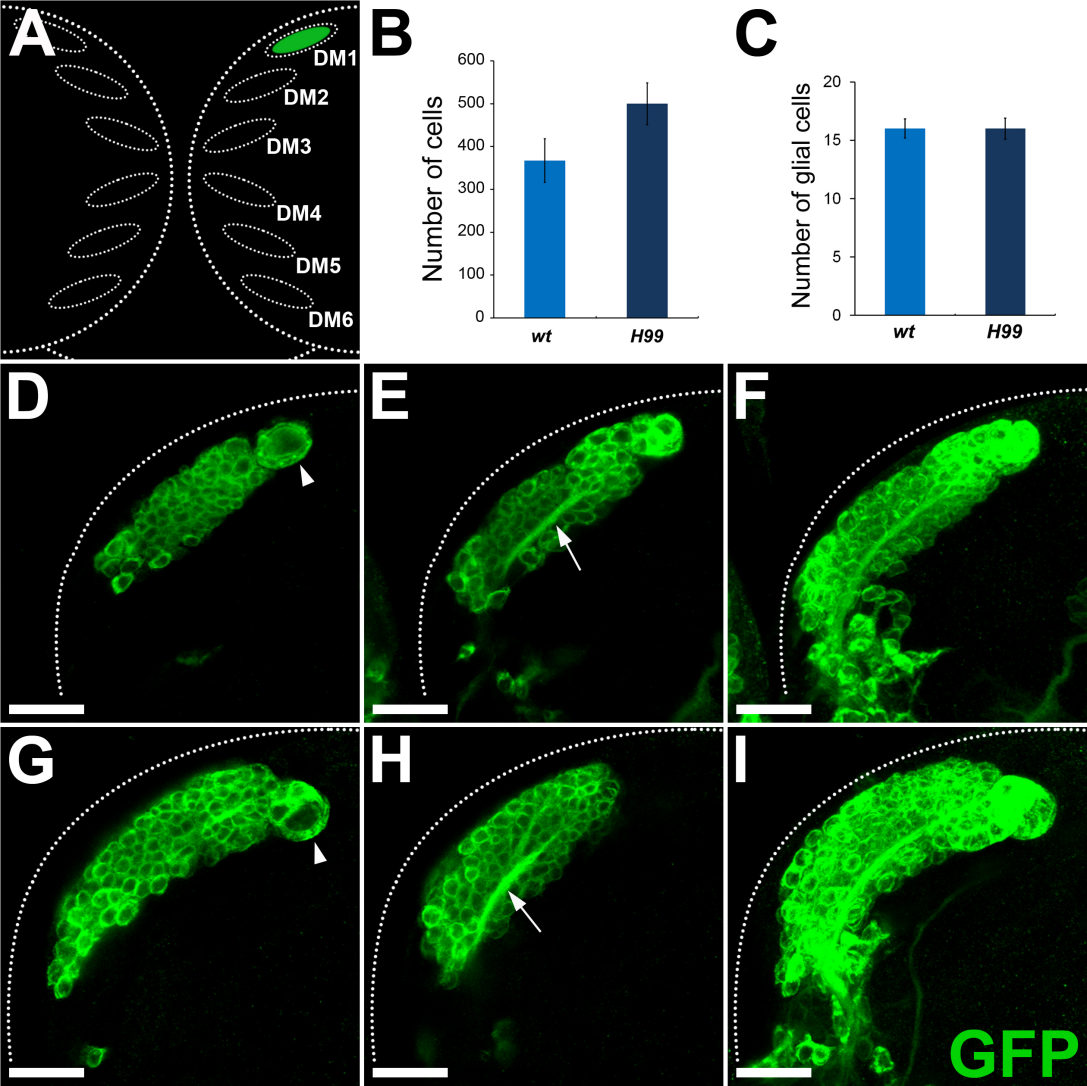
**Clonal analysis of apoptosis-blocked dorsomedial (DM) lineages during larval development**. **(A) **Schematic representation of the six DM lineages located at the posterior dorsomedial edge of the brain hemisphere. Cell number was quantified for the DM1 lineage (marked in green). **(B) **Average number of cells in wild-type (light blue) and apoptosis-blocked (dark blue) DM1 lineage. **(C) **Average number of glial cells in wild-type (light blue) and apoptosis-blocked (dark blue) DM1 lineage. **(D-F) **A wild-type mosaic analysis with a repressible cell marker (MARCM) clone of a DM1 lineage; cells labeled with mCD8::GFP (green). Single confocal sections at the level of (D) the neuroblast (arrowhead), and (E) the secondary axon tract (arrow), and superimposition of all single optical sections containing (F) labeled cells are shown. **(G-I) **A MARCM clone of a cell death-blocked DM1 lineage; cells labeled with mCD8::GFP (green). Single confocal sections at the level of (G) the neuroblast (arrowhead), and (H) the secondary axon tract (arrow), with (I) superimposition of all single optical sections containing labeled cells are shown. **(B, C) **Error bars represent standard deviation. Scale bars = 20 μm. Genotypes: **(D-F) ***hs-flp; tub-Gal4 UAS-mCD8::GFP; FRT2A tub-Gal80/FRT2A*; **(G-I) ***hs-flp; tub-Gal4 UAS-mCD8::GFP; FRT2A tub-Gal80/FRT2A H99*.

To confirm and quantify these findings, we carried out a MARCM-based clonal analysis of individual DM lineages. Lineage-specific apoptosis block was achieved by generating *H99 *mutant MARCM neuroblast clones. In the *H99 *mutant, the pro-apoptotic genes *hid *(*head involution defective*), *grim*, and *rpr *(*reaper*) are absent as a result of a homozygous deficiency *Df3(3L)H99 *in the genomic region at 75C [36-38]. Wild-type and apoptosis-blocked neuroblast clones were induced with ubiquitous *tubulin-Gal4 *driving *UAS-mCD8::GFP*. Clones were induced at larval hatching and recovered at the wandering third larval instar. Labeled wild-type and mutant neuroblast clones of lineages DM1 to DM6 were identified based on their size and relative position in the larval brain [12,30]. Although labeled wild-type and apoptosis-blocked mutant clones were recovered for all six DM lineages, we focused our analysis on the DM1 lineage because this lineage has a spatial arrangement of cell bodies that facilitates cell counts, and is also easiest to identify based on its position in the brain hemisphere (Figure 3A).

The overall organizational features of the wild-type DM1 lineage were documented in single optical sections of the MARCM labeled clones. A single large cell corresponding to the DM1 neuroblast was associated with a large cluster of labeled neural progeny (Figure 3D, arrowhead). Axons from these neural progeny fasciculated to form a secondary axon tract (SAT), which started within the cluster of labeled cells (Figure 3E, arrow). The overall extent of the labeled clone, shown by a superimposition of all single optical sections containing labeled cells, had the typical size and position of the wild-type DM1 lineage as reported by previous studies (Figure 3F) [12,30]. When corresponding single sections were documented for cell-death-blocked DM1 mutant clones, marked increases in the number of labeled cells were seen. This was especially evident in single optical sections taken at the level of the mutant DM1 neuroblast (Figure 3G, arrowhead; compare with Figure 3D). However, an increase in the number of labeled cells in mutant clones was also apparent in single sections taken at the level of the SAT (Figure 3H, arrow; compare with Figure 3E). Correspondingly, superimposition of all optical sections containing labeled cells showed a marked increase in size and cell number in the labeled apoptosis-blocked mutant DM1 clone (Figure 3I; compare with Figure 3F). The thickness of the SAT was increased in the apoptosis-blocked mutant MARCM clone, whereas the projection pattern of the SAT at the end the late third larval instar was the same as that seen in wild-type MARCM clones, and no ectopic axon projections were formed in the larval brain ([12,30]; data not shown).

To quantify the increase in cell number, we counted the number of labeled cells in a series of wild-type versus apoptosis-blocked mutant DM1 neuroblast clones (Figure 3B). Wild-type DM1 clones (n = 6) had an average of 367 ± 51 labeled cells, whereas the apoptosis-blocked mutant DM1 clones (n = 11) had an average of 500 ± 49 labeled cells. These findings indicate that 27% of the cells generated in DM1 clones are eliminated by apoptosis during larval development. To determine if the affected cells in the DM1 lineage were neurons or glial cells, we labeled wild-type and apoptosis-blocked mutant neuroblast clones with the glia-specific anti-Repo antibody, which reliably recognizes the glial cells in DM clones [30]. Counts of the number of the Repo-labeled cells did not reveal a difference in glial cell number in wild-type DM1 clones (16 ± 0.8; n = 4) versus mutant DM1 clones (16 ± 0.9; n = 7) (Figure 3C), indicating that the cells affected by apoptosis in DM1 lineages were neuronal but not glial cells. We conclude that programmed cell death eliminates approximately one-quarter of the neurons generated in the DM1 lineage during larval development.

Because the neural progeny in type II lineages are generated through INPs, the programmed cell death seen during larval development could be due to the loss of either the entire INP clonal population or to subsets of neurons in a given INP clone. To investigate this, we generated wild-type and cell-death-blocked MARCM-labeled INP clones, and determined the number of labeled cells in each INP clone. Clones were induced at larval hatching, and recovered at the wandering third larval instar. In wild-type INP clones, two to three neurons and two to three glial cells were seen (Table 1; see Additional file, Figure 1A, A'). However, apoptosis-blocked mutant INP clones consisted of six to seven neurons and two to three glial cells (Table 1; see Additional file, Figure 1B, B'). Blocking cell death in the INP clones consistently resulted in a marked increase of approximately 40% in cell number compared with the wild-type (Table 1). Moreover, this increase in cell number was due to an increase in neuronal cells; the number of glial cells was not affected by cell death block. These results indicate that a significant subset of the neuronal cells in the INP sublineages of type II DM lineages are eliminated by programmed cell death.

**Table 1:** Cell number in wild-type and apoptosis-blocked intermediate neural progenitor clones (e.g. H99)

	Total	Neuron	Glia
*Wild-type *(n = 4)	5.3 ± 0.5	2.5 ± 0.6	2.8 ± 0.5

*H99 *(n = 4)	9 ± 0.8	6.5 ± 0.6	2.5 ± 0.6

Although additional programmed cell death occurs during pupal development (Figure 2), we were not able to reliably identify DM1 neuroblast clones in the pupal stages because the lineage-related neurons changed position and no longer formed compact groups during metamorphosis [12]. Consequently, we could not accurately determine the number of cells that underwent programmed cell death in the DM1 lineage during pupal development.

### Blocking programmed cell death results in central complex neuropile innervation defects of DM lineage neurons

Our findings indicate that a significant level of programmed cell death occurs in the neural cells of DM lineages during normal development. To investigate the role of the normally occurring programmed cell death, we used lineage tracing to assay the contribution of wild-type DM lineages versus apoptosis-blocked DM lineages to the major neuropile compartments of the central complex in the adult brain. In these experiments, we combined synaptic neuropile labeling using the nc82 antibody with DM neuron-specific labeling using the *erm-Gal4^R09D11 ^*lineage-tracing method (see above).

**Figure 4:**
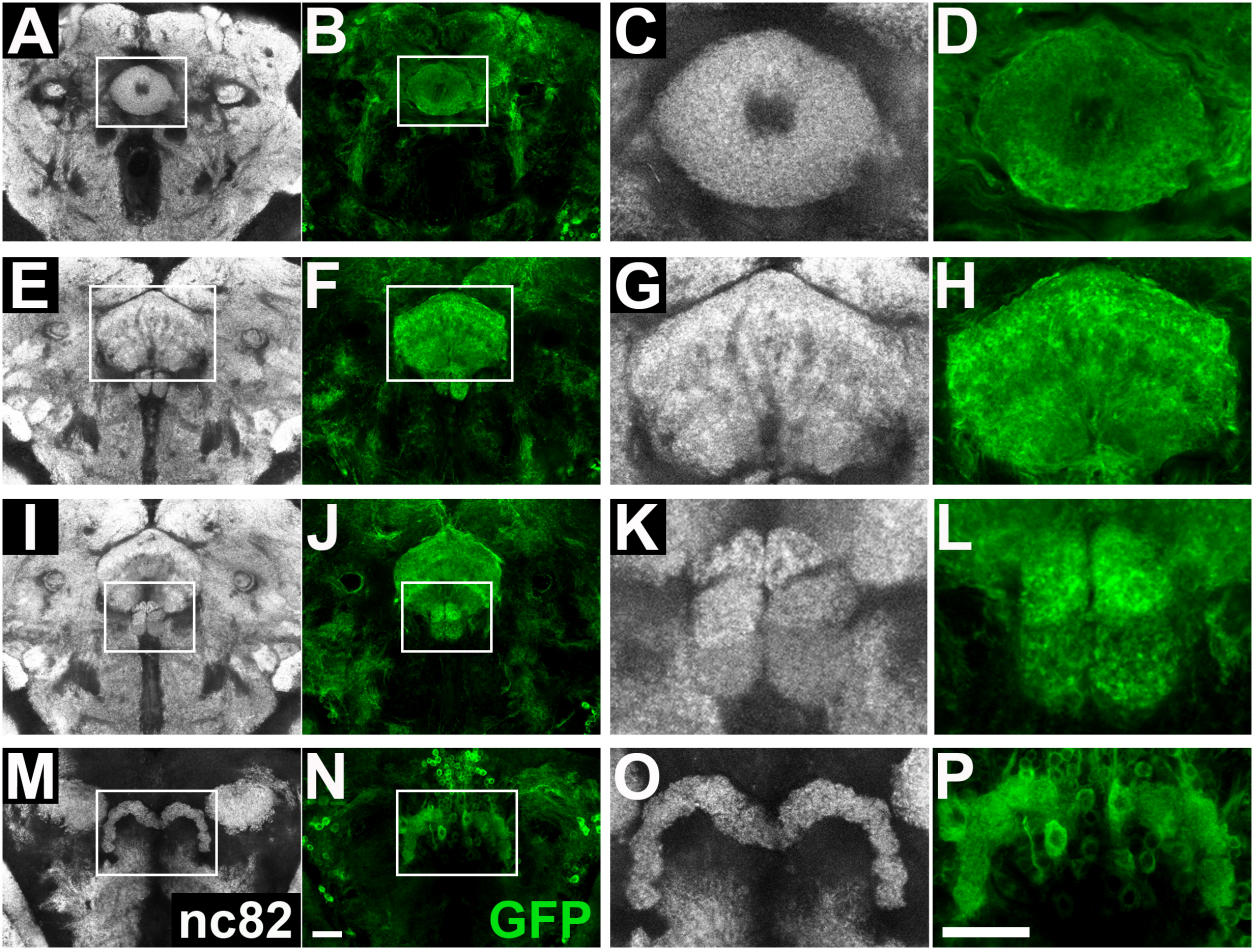
**Innervation of the central complex from wild-type dorsomedial (DM) lineages**. Confocal sections show the neuropile (white, labeled with nc82) and neuronal innervation of the central complex from wild-type DM lineages (green, labeled with mCD8::GFP) in adult brains at the level of **(A-D) **the ellipsoid body, **(E-H) **the fan-shaped body, **(I-L) **the noduli, and **(M-P) **the protocerebral bridge of the central complex. **(A, B, E, F, I, J, M, N) **Overview of the neuropile and the central complex innervation from DM lineages. **(C, D, G, H, K, L, O, P) **Close-up view of the central complex neuropile and the neuronal innervation from DM lineages. Scale bars = 20 μm. Genotype: *UAS-flp; erm-Gal4^R09D11 ^act>CD2>Gal4 UAS-mCD8::GFP*.

**Figure 5:**
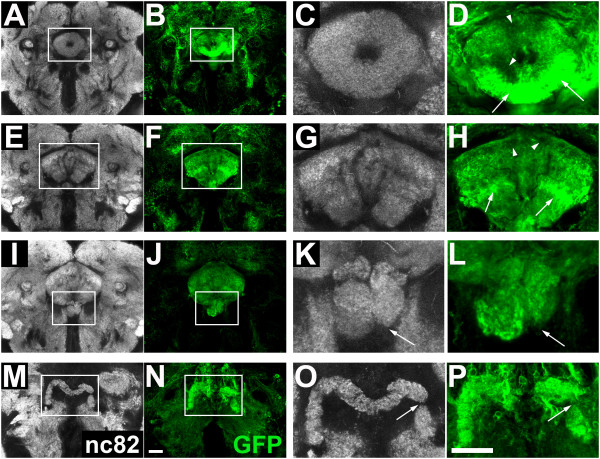
**Innervation of the central complex from apoptosis-blocked dorsomedial (DM) lineages**. Confocal sections show the neuropile (white, labeled with nc82) and neuronal innervation of the central complex from apoptosis-blocked DM lineages (green, labeled with mCD8::GFP) in adult brains at the level of **(A-D) **the ellipsoid body, **(E-H) **the fan-shaped body, **(I-L) **the noduli, and **(M-P) **the protocerebral bridge of the central complex. **(A, B, E, F, I, J, M, N) **Overview of the neuropile and the central complex innervation from apoptosis-blocked DM lineages. **(C, D, G, H, K, L, O, P) **Close-up view of the central complex neuropile and the neuronal innervation from DM lineages. Arrows and arrowheads indicate misinnervation by labeled neuronal projections and misarrangement of neuropile in the central complex (see text for details). Scale bars = 20 μm. Genotype: *UAS-flp; UAS-p35; erm-Gal4^R09D11 ^act>CD2>Gal4 UAS-mCD8::GFP*.

Neuropile-specific nc82 immunolabeling identified the EB of the wild-type brain as a prominent ring-shaped neuropile structure located anterior to the FB neuropile (Figure 4A, C). Labeling of the neuronal arborizations of the wild-type DM lineages in the EB showed prominent innervation of the entire ellipsoid body, which was characterized by a regular and relatively invariant degree of intensity throughout the ring-like neuropile (Figure 4B, D). When apoptosis was blocked in DM lineages, the general form of the EB compartment, as shown by nc82 labeling, was similar to that of the wild-type; however, some irregularity in the intensity of labeled neuropile was apparent (Figure 5A, C). Notably, labeling of the neuronal projections of the apoptosis-blocked DM lineages revealed the presence of abnormal arborizations (Figure 5B, D). Thus, labeled neuronal processes were seen to aggregate strongly in some parts of the EB ring (Figure 5D, arrows) and were relatively weak in other parts (Figure 5D, arrowheads). This type of perturbed innervation contrasted with the relatively uniform innervation seen in wild-type DM lineages (Figure 4D).

The neuropile of the FB is located posterior to the ellipsoid body and represents the largest central complex compartment of the *Drosophila *brain. In the wild-type brain it is subdivided into several regularly vertical and horizontal strata (Figure 4E, G). Labeling of the neuropile innervation from wild-type DM lineages identified a major contribution to the FB neuropile, as virtually all strata, either horizontal or vertical, received ordered innervation (Figure 4F, H). When apoptosis was blocked in DM lineages, the overall organization of the FB compartment as shown by nc82 labeling appeared normal, but some regions within the compartment appeared to lack synaptic neuropile (Figure 5E, G). Specific labeling of the neuronal innervation from the apoptosis-blocked DM lineages was clearly abnormal (Figure 5F, H), with lack of innervation in some strata (Figure 5H, arrowheads) and aberrant concentration of arborizations in others (Figure 5H, arrows). As in the case of the EB, this type of perturbed innervation in the FB contrasted with the relatively uniform innervation seen in wild-type DM lineages (Figure 4H).

The neuropile of the two bilaterally symmetrical NO is located ventrally to the FB, and is composed of three distinct and regularly arranged layers, as shown in the nc82-labeled wild-type brain (Figure 4I, K). The NO showed innervation from the wild-type DM lineages in all three layers (Figure 4J, L). In brains with apoptosis-blocked DM lineages, although NO were present, they were often misarranged (Figure 5I, K). Thus, nc82 labeling identified deformation in size and position of the three-layered structure of the neuropile (Figure 5K, arrow). Labeling of innervation from the apoptosis-blocked DM lineages reflected (and was probably the cause of) this dramatic misarrangement (Figure 5J, L). For instance, the bilaterally symmetrical organization of the three layers was lost in these brains (Figure 5L, arrow).

The neuropile of the m-shaped PB is located in the most posterior position relative to other central complex compartments (Figure 4M, O). It is divided into 16 segmental units, but these are revealed only poorly by nc82 labeling in wild-type brain. The two halves of the PB neuropile were innervated in a regular manner by lineage-specific labeled processes from the wild-type DM lineages, but this labeling was somewhat obscured because of the proximity of labeled cell bodies of the neurons projecting to the protocerebral bridge (Figure 4N, P). In brains with apoptosis-blocked DM lineages, the PB was present, but perturbations in the nc82 labeled neuropile of the protocerebral bridge were evident as breaks or deletions (Figure 5M, O, arrow). These breaks and deletions in PB innervation appeared to be amplified in specific labeling of projections from the apoptosis-blocked DM lineages (Figure 5N, P, arrow). Taken together, these findings indicate that abnormal innervation of major compartments of the central complex occurs specifically for the neurons from apoptosis-blocked DM lineages. In approximately one-third of the mutant flies, apoptosis blockage of DM lineages resulted in abnormal innervation of all four major central complex neuropile compartments, and in another third of the mutant flies, three of the four major central complex compartments had abnormal innervation (see Additional file, Table 1). We conclude that the extensive programmed cell death of neurons that occurs in DM lineages is essential for correct innervation of the central complex neuropile by these lineages.

In contrast to the abnormal innervation of neuropile compartments by arborizations of neurons from apoptosis-blocked DM lineages, the axonal projections of these neuronal lineages from their cell body clusters to the adult central complex neuropile were normal (data not shown). Moreover, all mutant flies were viable and fertile, and no obvious behavioral consequences of apoptosis blockage in DM lineages were seen; mutant flies had normal walking and climbing behavior in simple negative geotaxis and positive phototaxis assays.

### Development of central complex innervation defects in apoptosis-blocked DM lineage neurons

During the first two days of pupal development, the primordium of the central complex compartments becomes recognizable, undergoes characteristic shape changes, and grows in size as a result of the lineages that innervate the central complex form terminal arbors in the neuropile during this time [15]. To investigate the role of the normally occurring programmed cell death during this process, we used lineage tracing to characterize the projections of wild-type DM lineages versus apoptosis-blocked DM lineages to the primordium of the central complex in the pupal brain.

**Figure 6:**
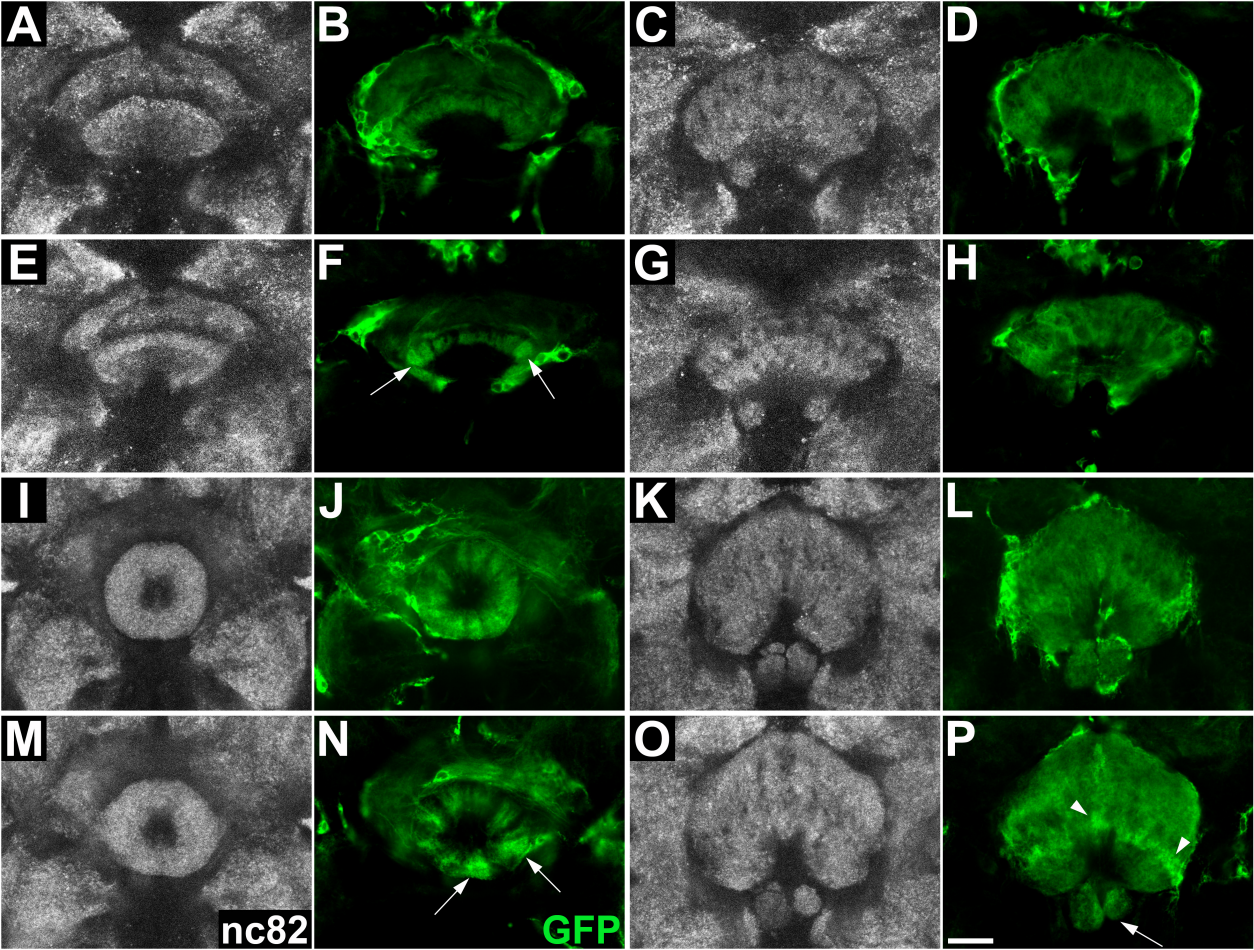
**Development of central complex innervation defects in apoptosis-blocked dorsomedial (DM) lineage neurons**. **(A-D) **Confocal images of single sections at the level of (A, B) the ellipsoid body and (C, D) the fan-shaped body in wild-type pupal brains at 24 hours after puparium formation (APF). Neuropile labeled with (A, C) nc82 and (B, D) neuronal innervation from DM lineages labeled with mCD8::GFP. **(E-H) **Confocal images of single sections at the level of (E, F) the ellipsoid body and (G, H) the fan-shaped body in the pupal brains of DM-specific apoptosis-blocked flies at 24 hours APF. (E, G) Neuropile labeled with nc82 and (F, H) neuronal innervation labeled with mCD8::GFP. (F) Perturbations in the innervation of the developing EB are indicated by arrows. **(I-L) **Confocal images of single sections at the level of (I, J) the ellipsoid body and (K, L) the fan-shaped body and noduli in pupal brains of wild-type at 48 hours APF. Neuropile labeled with (I, K) nc82 and (J, L) neuronal innervation labeled with mCD8::GFP. **(M-P) **Confocal images of single sections at the level of (M, N) the ellipsoid body and (O, P) the fan-shaped body and noduli in pupal brains of DM-specific apoptosis-blocked flies at 48 hours APF. Neuropile labeled with (M, O) nc82 and (N, P) neuronal innervation labeled with mCD8::GFP. Misinnervation and misarrangement in the developing central complex are indicated by (N, P) arrows and arrowheads (see text for details). Scale bar = 20 μm Genotypes: **(A-D, I-L) ***UAS-flp; erm-Gal4^R09D11 ^act>CD2>Gal4 UAS-mCD8::GFP ***(E-H, M-P) ***UAS-flp; UAS-p35; erm-Gal4^R09D11 ^act>CD2>Gal4 UAS-mCD8::GFP*.

At 24 hours APF, the wild-type central complex primordium was a bent, multilayered structure composed of the FB primordium with the primordia of the NO attached to its ventrolateral tips, and the primordium of the EB located ventrally as a slender crescent-shaped layer (Figure 6A, C). Labeled projections from wild-type DM lineages innervated all of these primordia in a regular manner (Figure 6B, D) (they also innervated the primordium of the PB; data not shown). At 24 hours APF, the central complex primordium in pupal brains with apoptosis-blocked DM lineages was comparable with that of wild-type brains in terms of the general appearance and arrangement of the primordia of the EB, FB, and NO (Figure 6E, G). Moreover, the labeled processes from apoptosis-blocked DM lineages innervated all of these primordia, albeit with some initial signs of abnormal innervation (Figure 6F, H); see, for example, those visible in the EB primordium (Figure 6F, arrows).

At 48 hours APF, the wild-type central complex primordium was already comparable with that of the mature adult structure (Figure 6I, K). The primordium of the EB had closed to form its characteristic ring-shaped appearance (Figure 6I), the FB primordium had the characteristic size and shape of the adult structure (Figure 6K), and the primordia of the NO formed a bilaterally symmetrical multilayered neuropile flanking the midline (Figure 6K). Labeled neuropile processes from wild-type DM lineages innervated all of these primordia in a regular manner, and this innervation often revealed subcompartments such as in the EB primordium (Figure 6J, L). The general arrangement of the central complex primordium at 48 h APF in pupal brains with apoptosis-blocked DM lineages was comparable with that of wild-type brains, and the primordia of all central complex subcompartments were evident (Figure 6M, O). However, the labeled neuropile processes from apoptosis-blocked DM lineages showed clear signs of abnormal innervation in these primordia (Figure 6N, P). For example, labeled processes in the EB were seen to aggregate strongly in some parts of the ring-shaped compartment (Figure 6N, arrows) and the FB primordium (Figure 6P, arrowheads), and misarrangement of the layers of the NO neuropile was also seen in these brains (Figure 6P, arrow).

We conclude that initial signs of abnormal innervation from apoptosis-blocked DM lineages are visible in the developing central complex primordium at 24 hours APF, and that prominent abnormal innervation by these apoptosis-blocked lineages becomes apparent at 48 hours APF. We posit that the initial defects in innervation in the early pupal brain form the basis for the severe abnormal innervation that characterizes apoptosis-blocked DM lineages in the adult central complex.

## Discussion

In this study, we used a combination of genetic lineage tracing, clonal MARCM techniques, and molecular labeling to study programmed cell death in the DM type II neuroblast lineages that make major contributions to the central complex neuropile. Our data indicate that extensive programmed cell death occurs in these lineages during post-embryonic development, and that this lineage-specific cell death is important for the establishment of appropriate innervation of the DM lineage-derived neurons in the adult central complex. These findings have implications for understanding the mechanisms that underlie type II neuroblast proliferation, post-embryonic neural development, and the formation of complex circuitry in the central brain.

Programmed cell death has been widely studied in development of the central nervous system of *Drosophila*. Most of these studies have focused on lineages in the developing ventral nerve cord, in which cell death controls neuroblast and neural cell survival, often in a segment-specific manner [18-22,26,39,40]. Recently, lineage-specific programmed cell death has been reported in central brain development, and related to specific sublineages. A study of the four Engrailed-expressing neuroblast lineages in the brain shows that apoptosis specifically affects the Engrailed-negative cells in two lineages and the Engrailed-positive cells in another lineage in a process that is regulated by Notch signaling [24]. A comparable study of three identified neuroblast lineages that innervate the antennal lobe indicates that programmed cell death can target individual hemilineages in a Notch/Numb-dependent manner [25]. However, all of these investigations have been carried out on type I neuroblast lineages, and nothing was known about the existence of programmed cell death in the novel type II neuroblast lineages that amplify proliferation through INPs.

The experiments reported here indicate that programmed cell death plays a prominent role in the development of type II lineages. This means that the same single neuroblast lineage initially amplifies the number of neuronal cells through transit amplifying cell-like INPs, and subsequently reduces the number of neuronal cells that survive through apoptosis. This suggests that programmed cell death in these lineages may contribute to the balance between self-renewal and differentiation, which might otherwise contribute to depletion or overproliferation in these lineages [7,8]. Notably, uncontrolled overproliferation in these amplifying neuroblast lineages can lead to dramatic defects. For example, dramatic overgrowth and tumor formation in the developing *Drosophila *brain can occur if any one of several key cell-fate determinants that are normally segregated from the type II neuroblasts to their INP daughter cells to ensure differentiation are perturbed [16,41,42]. Interestingly, approximately one-quarter of the adult-specific neurons in the DM lineages that are targeted by apoptosis are both generated and eliminated during larval development before their parent DM neuroblasts cease their proliferative activity. Because adult-specific neurons only differentiate fully to form functional interconnections during subsequent pupal development, the affected cells are probably eliminated before they form synapses and integrate into the functional circuitry. However, apoptosis in these lineages is not limited to larval stages; it also occurs during early pupal stages when adult-specific DM neurons arborize and form synaptic connections that give rise to mature neuropile structures such as the central complex. It will be important to investigate this pupal phase of DM neuron apoptosis further, because it may indicate that neurons that fail to form appropriate synaptic interconnections in the neuropile are targeted by programmed cell death.

Our findings indicate that programmed cell death in DM lineages plays an important role in determining the innervation of the DM-derived neurons in the central complex neuropile. In the absence of apoptosis, aberrant and often asymmetric arborizations from these neurons are formed in all of the major substructures of the central complex neuropile. Moreover, these innervation defects are often accompanied by prominent neuropile disruptions, such as malformation of the NO or breakage in the PB. Our data document the major contribution of DM lineage-derived neurons to the developing central complex, and confirm similar findings by previous authors [12,13,30]. It is likely that comparable lineages contribute to central complex formation in other insects. Recent work in the grasshopper *Schistocerca gregaria *showed that four identified neuroblasts that are similar to DM1 to DM4 in *Drosophila *in terms of location and type of proliferation, also generate the numerous small-field neurons of the central complex [43,44]. Moreover, the lineages that are generated by these four grasshopper neuroblasts are targeted by programmed cell death, resulting in approximately 20% of the neurons being eliminated during embryonic development [43]. These remarkable similarities in a holometabolic insect such as *Drosophila *and a hemimetabolic insect such as the grasshopper, which are separated by at least 300 million years of evolution, imply that comparable neuroblasts, neuronal proliferation modes, and lineage-specific programmed cell death operate in the development of the central complex in most, if not all, insects [14].

The insect central complex is an intricate, high-level, multimodal information- storage and -processing center that is involved in motor coordination of behaviors such as walking, flying and stridulation [45]. In neuroanatomical terms, it represents a highly complex midline neuropile made up of several thousands of neurons, corresponding to approximately 50 cell types that are arranged in a highly ordered architecture. Remarkably, the large number of neurons that contribute to the central complex is generated by a small number of neuroblasts during a short developmental time period. Indeed, the amplification of DM neuroblast proliferation through INPs, which act as transient amplifying cells, may be an important mechanistic prerequisite for rapidly generating the large number of neurons required for complex central circuitry. Comparable amplification of neural stem-cell proliferation through transit amplifying cells is present in the developing brains of vertebrates including mammals. Notably, in mammalian cortical development, the wealth of neurons required for complex circuitry is largely generated by neural stem cells via INP-like progenitor cells called basal progenitors [46-48]. Given that this type of amplification of neural proliferation may be a general strategy for increasing the size and complexity of the brain, a concurrent and potentially counter-balancing role of programmed cell death in neural stem-cell lineages may also be a general and evolutionarily conserved mechanism for generating brain complexity.

## Methods

### Fly strains

Fly strains were maintained on standard medium, and experiments were performed at 25°C unless noted otherwise. The stocks were used in this study:

• y w hs-flp; tub-Gal4 UAS-mCD8::GFP^LL5^/CyO act-GFP^JMR1^; FRT2A tub-Gal80^LL9^

• w; FRT2A

• w; FRT2A H99 kni^ri^-2/TM6B

• w; UAS-mCD8::GFP^LL5^; erm-Gal4^R09D11^

• y w UAS-flp; If/CyO y^+^; act>CD2>Gal4 erm-Gal4^R09D11 ^UAS-mCD8::GFP^LL6^/TM6B

• w; UAS-p35; erm-Gal4^R09D11 ^UAS-mCD8::GFP^LL6^

### MARCM analysis

To generate wild-type MARCM clones, virgin females of *y w hs-flp; tub-Gal4 UAS-mCD8::GFP^LL5^/CyO act-GFP^JMR1^; FRT2A tub-Gal80^LL9 ^*were crossed with *w; FRT2A *males. Homozygous H99 MARCM clones were generated by crossing virgin females of *y w hs-flp; tub-Gal4 UAS-mCD8::GFP^LL5^/CyO act-GFP^JMR1^; FRT2A tub-Gal80^LL9 ^*with *w; FRT2A H99 kni^ri^-2/TM6B *males. Embryos from the above crosses were collected on agar plates for 4 hours and raised at 25°C for 22 hours before undergoing heat-shock treatment. Heat-shock induction of Flippase was performed by immersing the agar plates in a water bath at 37°C for 60 minutes (for neuroblast clones) or 18 minutes (for INP clones). Larvae were collected and plated at low density on standard medium. MARCM clones were then recovered at the wandering third larval instar or at the adult stage.

### Apoptosis analysis

To determine when DM lineage-specific apoptosis occurs during larval development, embryos of *w; UAS-mCD8::GFP^LL5^; erm-Gal4^R09D11 ^*were collected on agar plates for 4 hours and raised at 25°C for 22 hours. Larvae were collected, and then raised on standard medium at 25°C for 48, 60, 72, 84, and 96 hours before their brains were dissected. To study apoptosis in pupal brains, white pupae of *w; UAS-mCD8::GFP^LL5^; erm-Gal4^R09D11 ^*were collected and raised at 25°C for 6, 12, 24, 32, 48, and 72 hours before brain dissection. Apoptotic cells were detected in these brains by immunostaining with anti-activated Caspase-3 antibodies.

### Immunohistochemistry and antibodies

Larval brains were dissected in ice-cold PBS and fixed in 2% paraformaldehyde (PFA) for 60 minutes at room temperature, washed several times in PBS/0.5% Triton X-100, and pre-incubated in 10% normal goat serum. Brains were incubated with primary antibodies overnight at 4°C, and with secondary antibodies for 2 to 3 hours at room temperature. Pupal brains were dissected in ice-cold PBS and fixed in 4% PFA for 30 minutes at room temperature, and washed several times in PBS/0.5% Triton X-100. Brains were incubated with primary and secondary antibodies, each for 24 hours at 4°C. Adult brains were dissected from 4 to 7-day-old females in ice-cold PBS and fixed in 4% PFA for 30 minutes at room temperature, and washed several times in PBS/0.5% Triton X-100. Brains were incubated with primary antibodies for 48 hours at 4°C, and with secondary antibodies for 48 hours at 4°C.

Primary antibodies used in this study were: chicken anti-green fluorescent protein (GFP) (1:1000; ab13970; Abcam, Cambridge, MA, USA); mouse anti-Repo (1:30; 8D12; Developmental Studies Hybridoma Bank (DSHB), Iowa City, IA, USA); mouse anti-neurotactin (1:20; BP106; DSHB); rat anti-Elav (1:30; DSHB); rabbit anti-activated Caspase-3 (Asp175) (1:100; Cell Signaling Technology, Beverly, MA, USA); mouse anti-nc82 (1:50; a gift from J. Pielage). Secondary antibodies were: Alexa 488-, Alexa 568-, and Alexa 647-conjugated anti-chicken, -rabbit, -rat, or -mouse IgG (all 1:500; all from Molecular Probes Inc., Eugene, OR, USA).

### Microscopy and image processing

Immunofluorescent images were recorded on a confocal microscope (TCS SP5; Leica, Heerbrugg, Switzerland), and processed using ImageJ and Photoshop (Adobe Systems Inc., San Jose, CA, USA) software. Cells representing DM1/2 lineages in wild-type and *H99 *MARCM clones were counted using the Cell Counter plugin for ImageJ (K. De Vos).

## List of abbreviations

ALH: after larval hatching; APF: after puparium formation; DM: dorsomedial; DSHB: Developmental Studies Hybridoma Bank; EB: ellipsoid body; FB: fan-shaped body; GFP: green fluorescent protein; GMC: ganglion mother cell; INP: intermediate neural progenitor; MARCM: mosaic analysis with a repressible cell marker; NO: noduli; PB: protocerebral bridge; PBS: phosphate-buffered saline; SAT: secondary axon tract.

## Competing interests

The authors declare that they have no competing interests.

## Authors' contributions

YJ carried out all the experiments. HR conceptualized the project. YJ and HR analyzed the data and wrote the manuscript. All authors have read and approved the final manuscript.
